# Supplementary material for: X-ray crystallographic fragment screening reveals novel and conformationally dynamic ligand-binding sites in Mycobacterium tuberculosis FtsZ
Source: bioRxiv. 2026 Sep 2:2026.09.01.748605. Preprint. [Version 1] doi: 10.64898/2026.09.01.748605 (PMC13684551; doi:10.64898/2026.09.01.748605)
Supplement: Supplementary file 1 [file NIHPP2026.09.01.748605V1-supplement-1.pdf]

bioRxiv preprint doi: https://doi.org/10.64898/2026.09.01.748605; this version posted September 2, 2026. The copyright holder for this preprint (which was not certified by peer review) is the author/funder. This article is a US Government work. It is not subject to copyright under 17 USC 105 and is also made available for use under a CC0 license.

Supplemental Figures

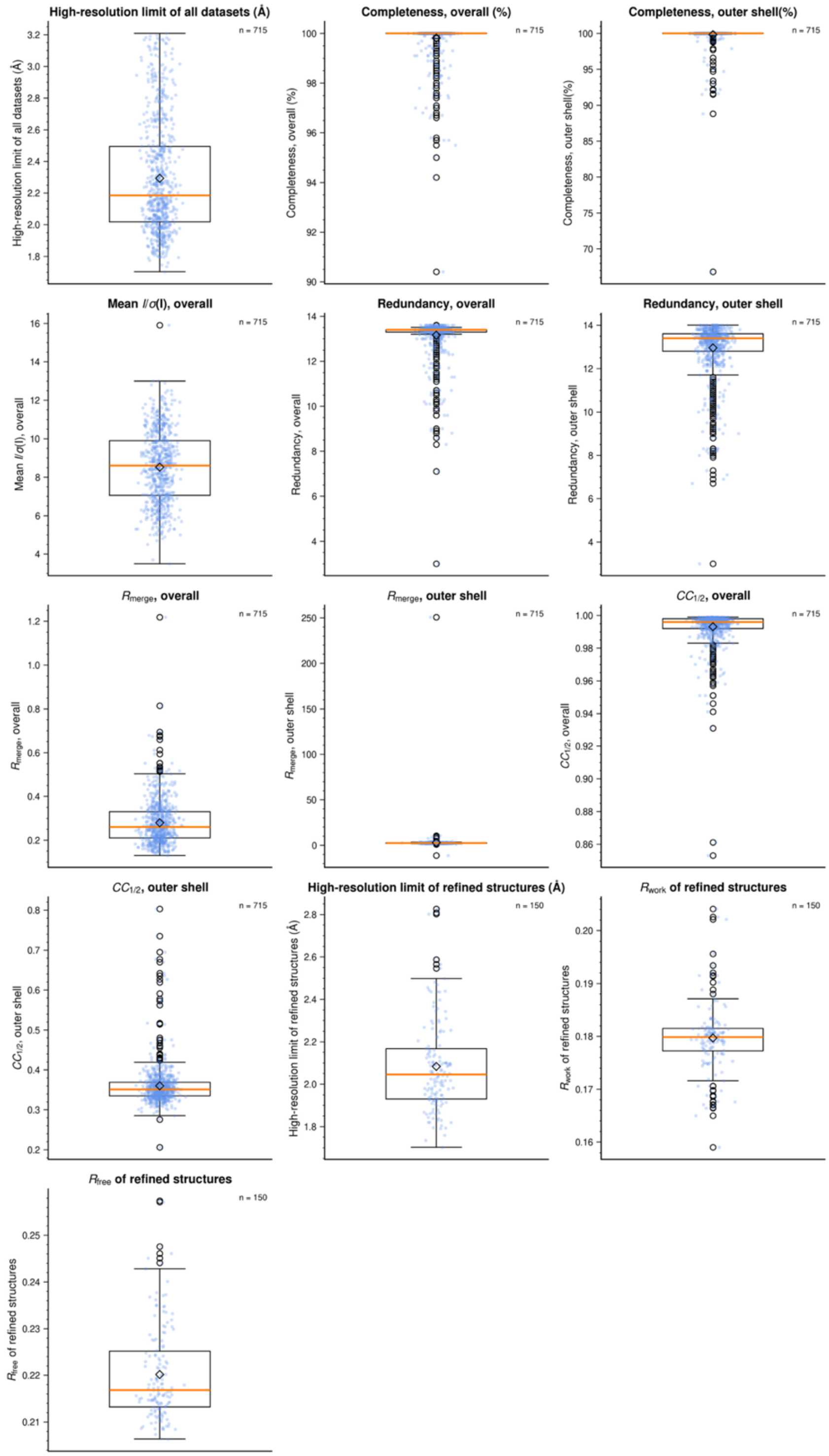

Figure S1. Data collection and refinement statistics shown as boxplots. The median for each parameter is represented by the orange line in the box. The mean is represented as the diamond. Outliers are shown in white circles.

bioRxiv preprint doi: https://doi.org/10.64898/2026.09.01.748605; this version posted September 2, 2026. The copyright holder for this preprint (which was not certified by peer review) is the author/funder. This article is a US Government work. It is not subject to copyright under 17 USC 105 and is also made available for use under a CC0 license.

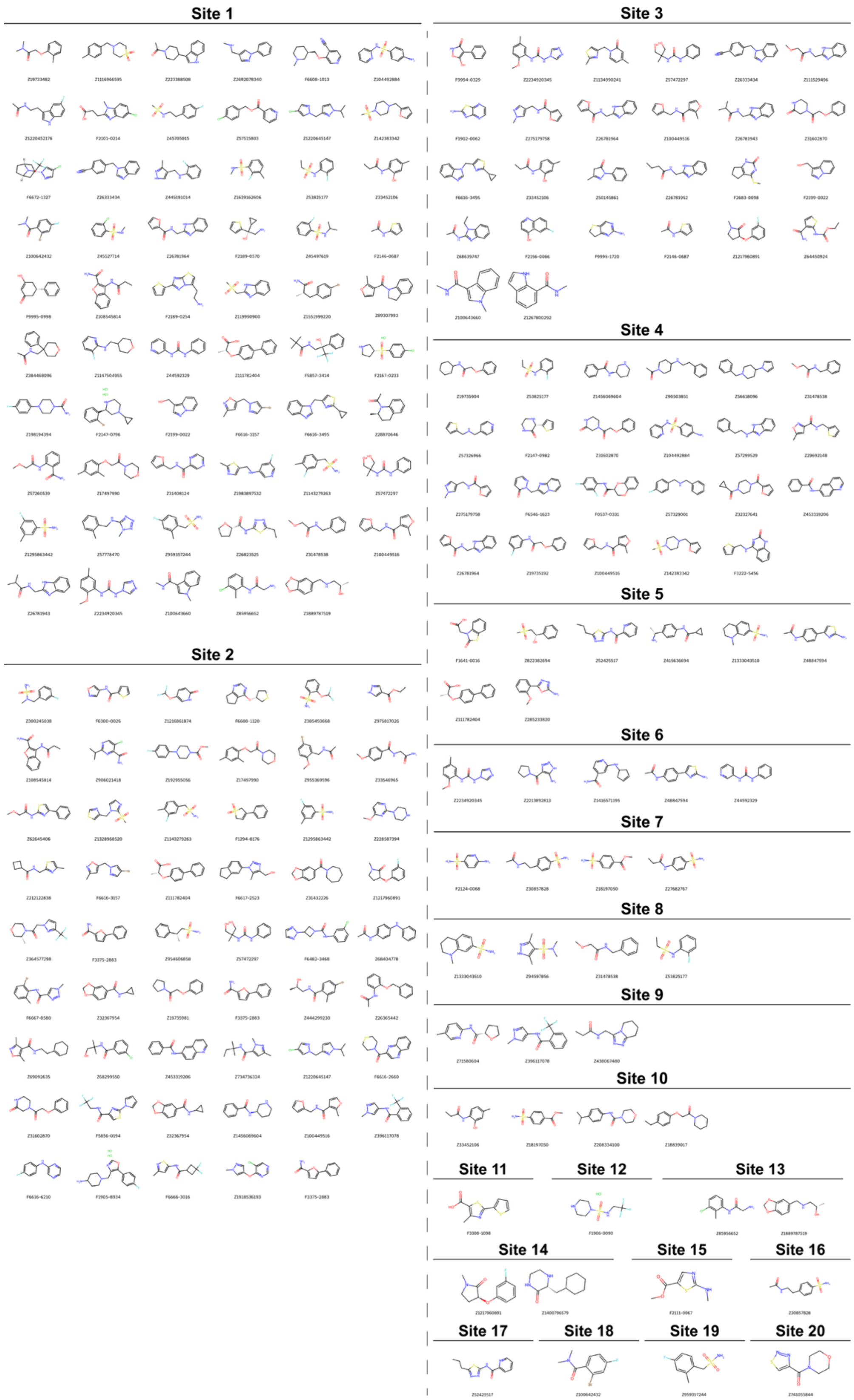

Figure S2. Chemical structure of hit fragments grouped by binding site.

bioRxiv preprint doi: https://doi.org/10.64898/2026.09.01.748605; this version posted September 2, 2026. The copyright holder for this preprint (which was not certified by peer review) is the author/funder. This article is a US Government work. It is not subject to copyright under 17 USC 105 and is also made available for use under a CC0 license.

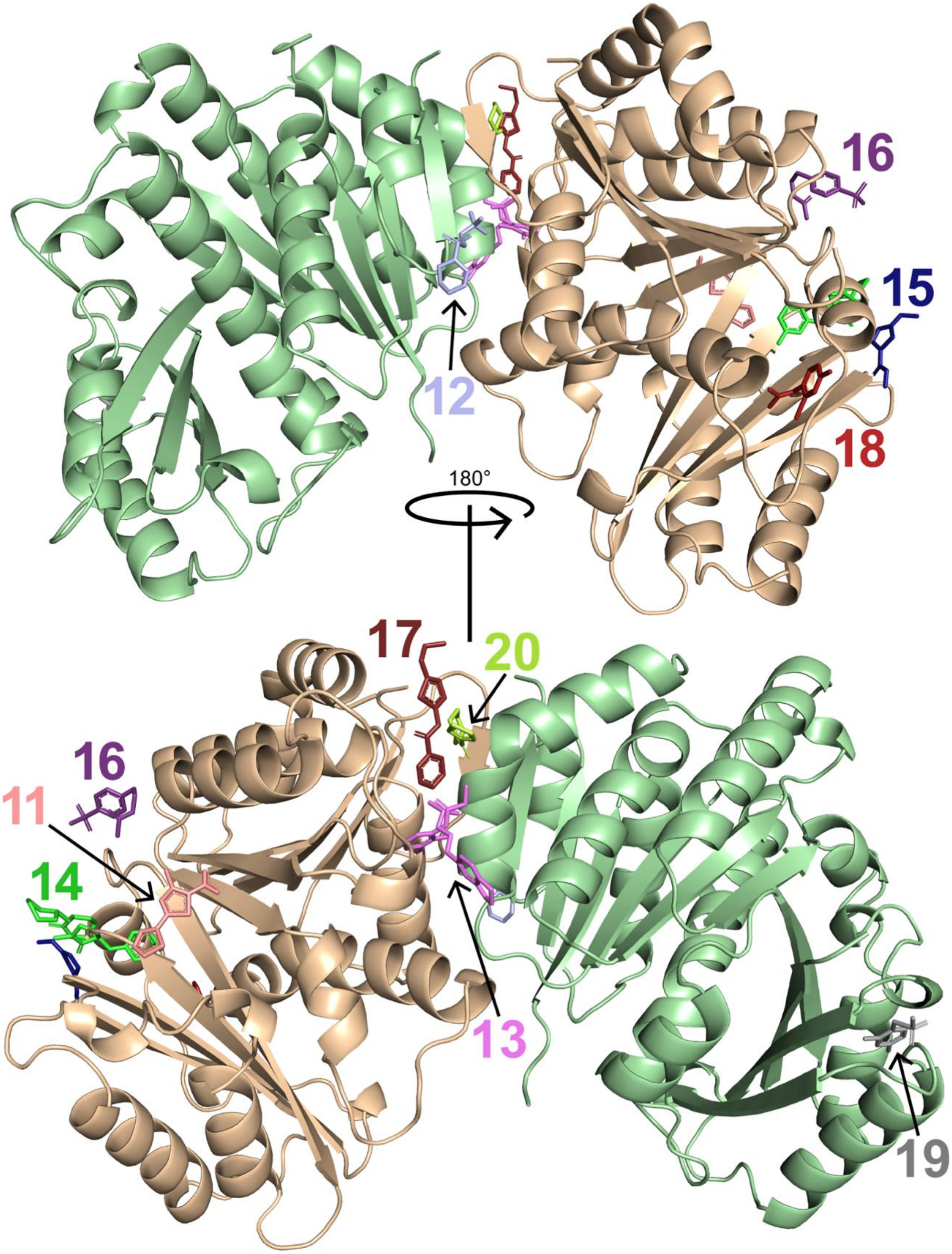

Figure S3. Locations of fragment-binding sites 11-20. Two views of the MtbFtsZ ground-state asymmetric unit are shown, related by a 180° rotation. Chain A (ON protomer) is pale green and chain B (OFF protomer) is wheat. Fragments are shown as sticks and colored according to sites ID.

bioRxiv preprint doi: https://doi.org/10.64898/2026.09.01.748605; this version posted September 2, 2026. The copyright holder for this preprint (which was not certified by peer review) is the author/funder. This article is a US Government work. It is not subject to copyright under 17 USC 105 and is also made available for use under a CC0 license.

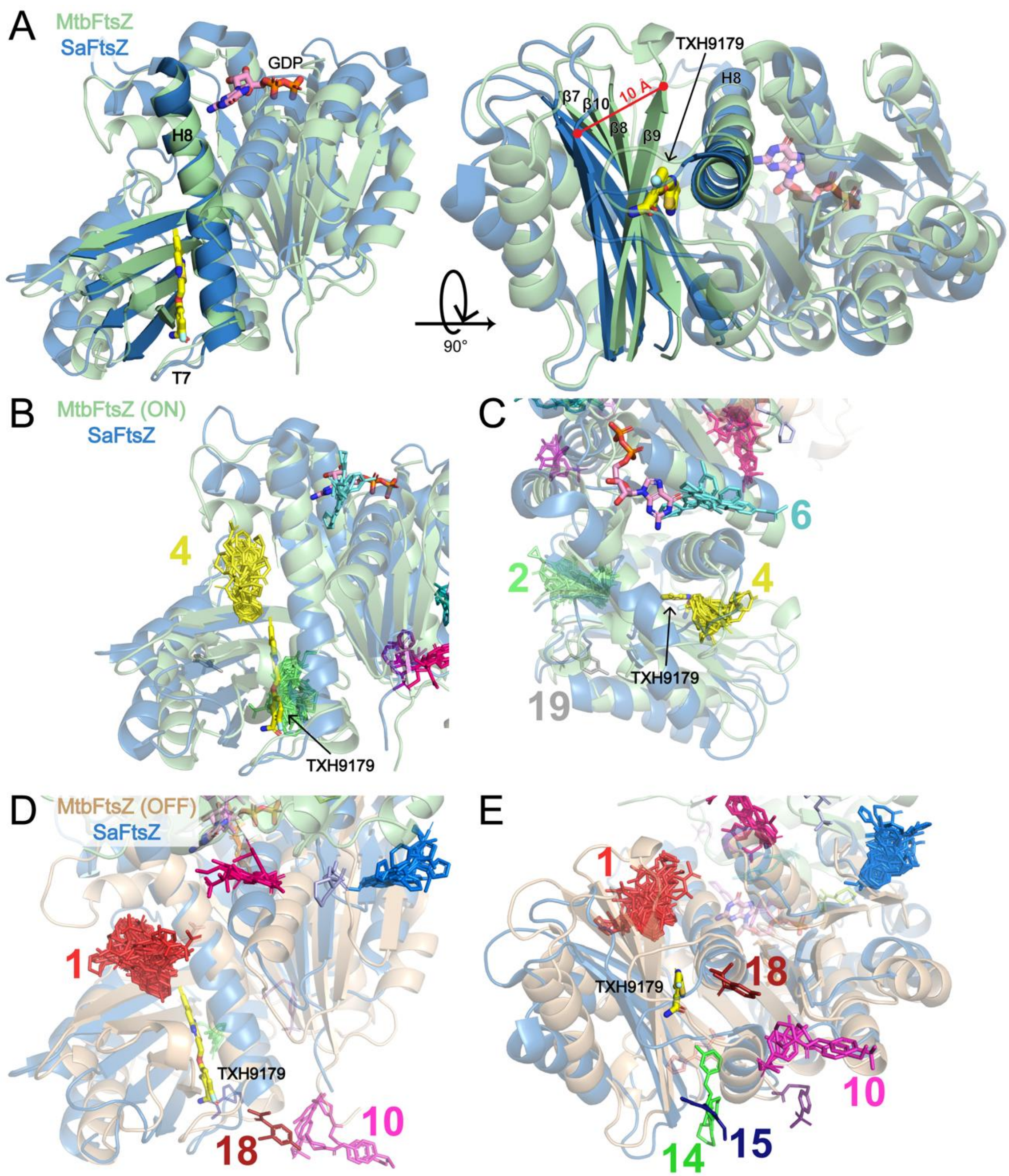

Figure S4. SaFtsZ (blue) allosteric binding pocket. **A**) Crystal structure of SaFtsZ complexed with TXH9179 (yellow) and GDP (pink) superposed to the ground state FtsZ model chain A (ON protomer; pale green). The structures are aligned by the core helix (H8 in MtbFtsz). **B-C**) SaFtsZ superposed onto MtbFtsZ chain A with hit fragments shown in sticks. **D-E**) SaFtsZ superposed onto the MtbFtsZ chain B (OFF protomer; wheat) with hit fragments shown in sticks.

bioRxiv preprint doi: https://doi.org/10.64898/2026.09.01.748605; this version posted September 2, 2026. The copyright holder for this preprint (which was not certified by peer review) is the author/funder. This article is a US Government work. It is not subject to copyright under 17 USC 105 and is also made available for use under a CC0 license.

Supplementary Table S1. PDB accession codes and crystallographic information for MtbFtsZ fragment-screening structures.

ftsz_supplemental_table1_20260824.csv

Each fragment-bound structure obtained from the crystallographic fragment is listed, the unmodelled datasets (pdb_000015aa) are listed last. For each structure, the screened fragment identifier, PDB chemical component ID, fragment-binding site(s), ligand chain and corresponding MtbFtsZ conformational state, refinement and crystallographic statistics are provided. Structures containing a fragment at more than one binding site list all observed sites and corresponding chain/state assignments. Enantiomers of fragments within the same dataset are separated into individual rows. All structures were deposited as part of PDB group deposition G_1002379.

Supplemental File 1. DNA sequence of pET-28b(+)_MtbFtsZ

pET-28b-plus_MtbFtsZ.txt
